# Penetrating Orbital and Intracranial Injury Due to a Ballpoint Pen: A Case Report

**DOI:** 10.7759/cureus.108700

**Published:** 2026-05-12

**Authors:** Arun Arumugam, Nadia Namous, David Baker, M. Salman Ali, Juhi Bansal

**Affiliations:** 1 Radiology, Augusta University Medical College of Georgia, Augusta, USA; 2 Neurological Surgery, Augusta University Medical College of Georgia, Augusta, USA; 3 Neurological Surgery, Wellstar Medical College of Georgia Health, Augusta, USA

**Keywords:** neuro-ophthalmology, neuroradiology, neurosurgery, penetrating injury, radiology

## Abstract

Penetrating head trauma represents a rare type of traumatic brain injury that is characterized by high mortality and morbidity. An orbitocranial penetrating injury presents with increased complexity due to its potential effects on structures of both the eye and brain, including the orbital apex, optic canal, and various lobes of the brain. This report considers the case of a 64-year-old male who suffered an injury caused by a ballpoint pen after an altercation. The injury was complicated by intracranial bleeding, seizures, and visual disturbances. Therefore, it required a multidisciplinary approach in ruling out potential severe complications of neurologic and visual character. The prompt evaluation and treatment helped address both immediate issues and the risks associated with possible development of long-term consequences such as visual impairments, paresis, and cognitive disorders. This case demonstrates the complexity of orbitocranial penetrating trauma and highlights the importance of a multidisciplinary collaboration and careful neuro-ophthalmologic assessment in optimizing patient outcomes.

## Introduction

Penetrating head trauma that traverses through both the skull and brain is a highly uncommon occurrence that is known to only contribute to 0.04% of all head traumas [1,2]. While intracranial injuries in comparison represent 24% of all penetrating head traumas, the occurrence of transorbital injuries that penetrate both the skull and brain is highly uncommon [3]. When penetrating head traumas as such occur, these injuries are known to be highly dangerous and have been known to have potential complications, including blindness, traumatic aneurysms, brain damage, and hypesthesia that requires immediate attention to prevent both long-term and fatal consequences [2,4]. Utilization of imaging is imperative to visualize the path of the penetrating injury and the structures the object may have come into contact with. From an ophthalmological standpoint, this includes structures such as the optic nerve, optic canal, and orbital apex. And when the penetrating trauma further includes the brain, this introduces several additional arteries and structures to consider with both acute and chronic impacts. Traumas of such nature can occur both unintentionally and intentionally in manners such as altercations or falls, and as such can present with patients of a wide range of ages and different past medical histories. The penetrating objects themselves are often taken out in the operating room due to consideration for large hemorrhaging [5,6]. This case presents a 64-year-old male who was struck by a ballpoint pen and illustrates the many considerations clinicians must undertake to ensure proper removal of the penetrating object while minimizing negative neurological and ophthalmological consequences acutely and chronically.

## Case presentation

**Figure 1:**
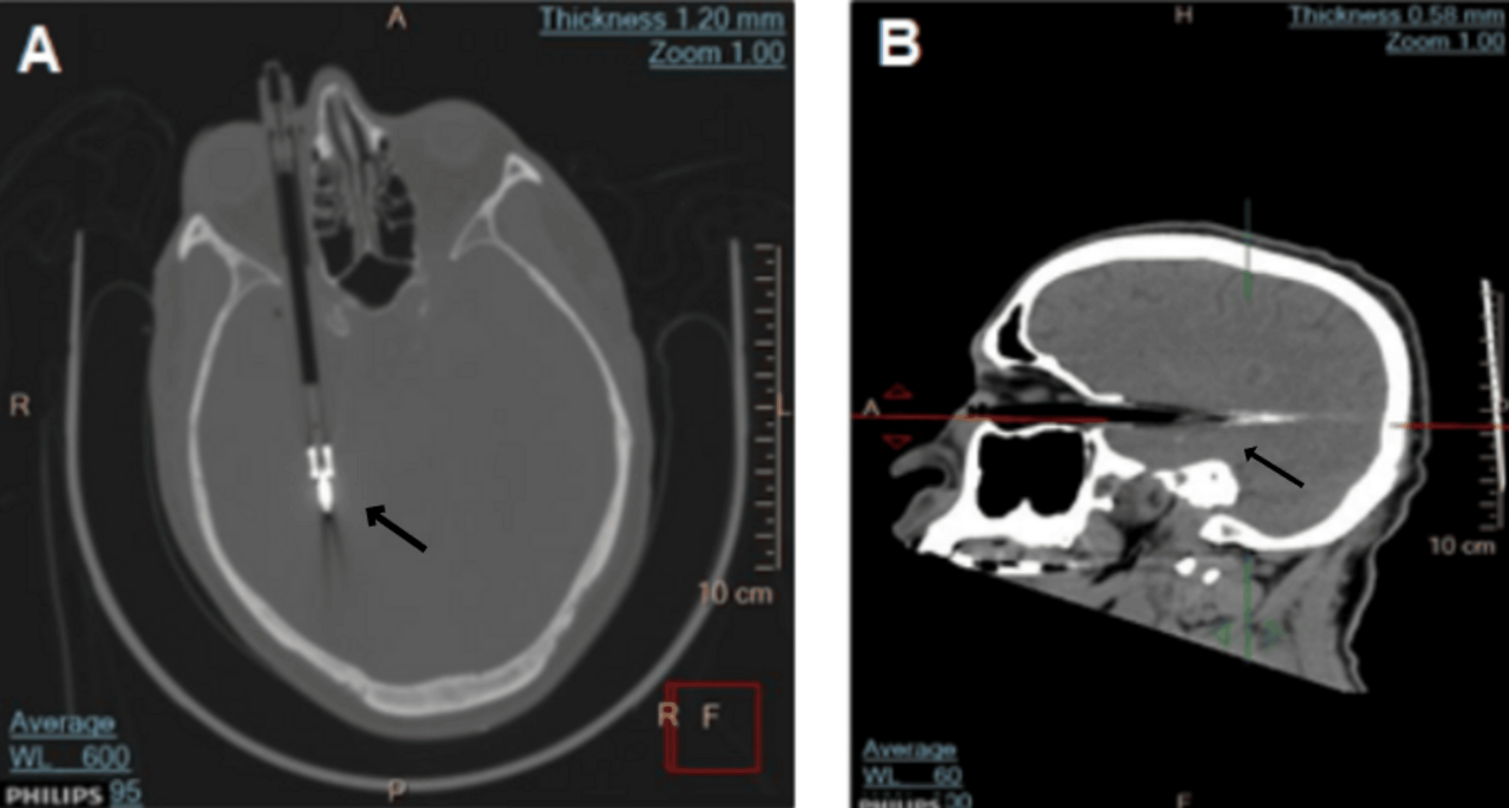
CT of the head outlining the extent of pen insertion. A. Axial CT scan of the head providing a more detailed view of the placement of the ballpoint pen through the right orbit and brain. B. Sagittal CT view of the head highlighting the level of penetration of the ballpoint pen through the right orbit and traversing into the right temporal lobe.

In November 2024, a 64-year-old male presented with a level 1 penetrating trauma to the right face/eye after being stabbed in the right eye with a ballpoint pen during an altercation. On arrival, the pen was still lodged below the right orbit, but the patient was alert and oriented to person, place, and time with a Glasgow Coma Scale (GCS) score of 14 [7]. The patient reported decreased visual acuity in the affected eye but was able to perceive shapes. There was no active bleeding present around the point of penetration. CT scans were taken afterwards to evaluate the placement of the ballpoint pen within the patient's skull (Figure 1).

The non-contrast CT views of the head and maxillofacial region demonstrated that the ballpoint pen traversed through the medial right globe, optic canal, and terminated in the posterior right temporal lobe, adjacent to the occipital horn of the lateral ventricle. Alongside the penetration, a small-volume subarachnoid hemorrhage, a tentorial subdural hematoma, and an intraparenchymal 3 x 4 mm right temporal lobe hematoma were noted along the path of the pen. Ophthalmology was consulted and determined that findings were consistent with optic nerve trauma due to the patient's poor vision, fixed and dilated pupil, and normal posterior exam.

**Figure 2:**
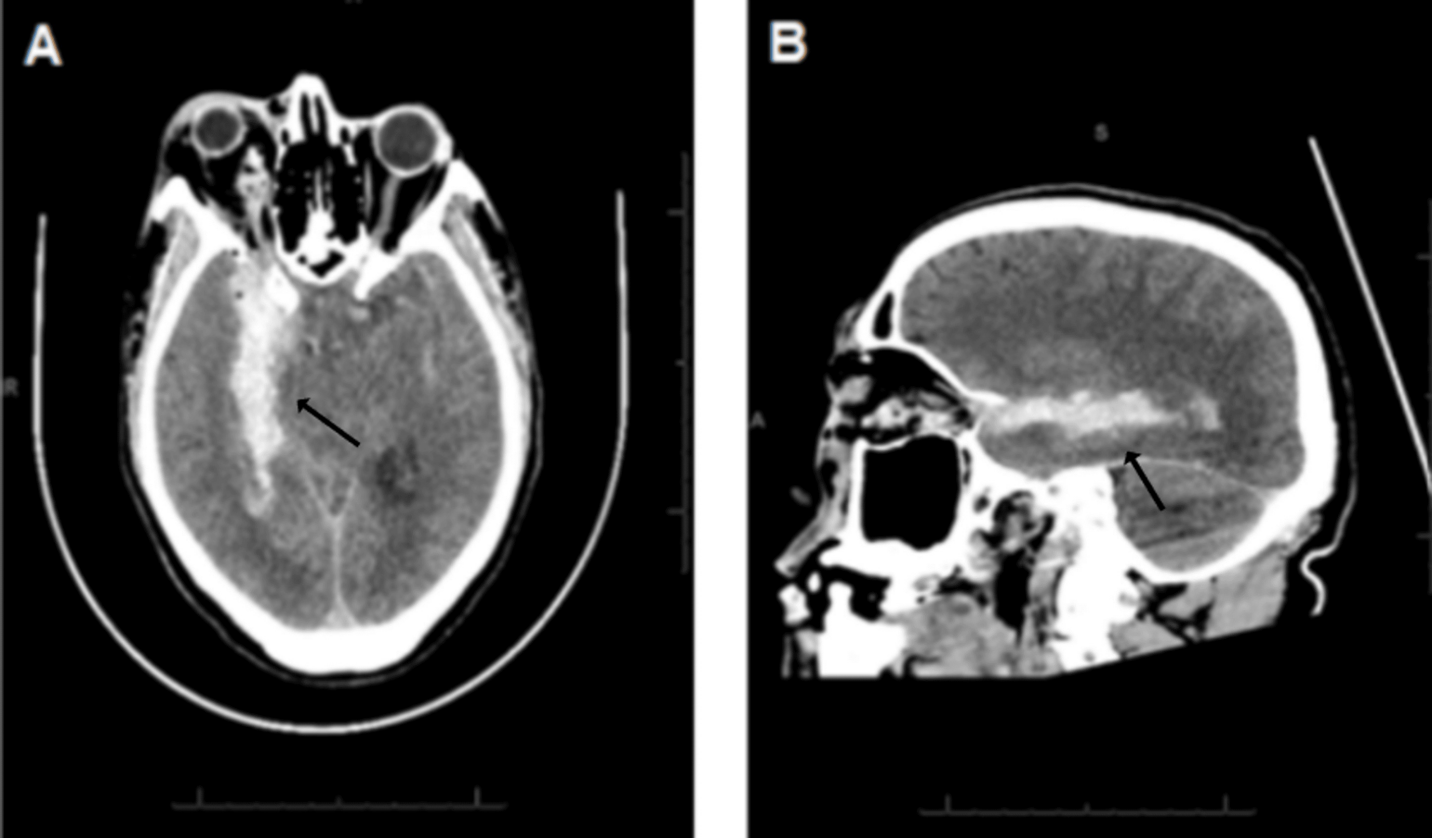
CT of the head after removal of the ballpoint pen. A. Axial CT scan of the head after removal of the pen showing the level of intracranial hemorrhage present.​ B. Sagittal CT scan status post pen removal demonstrated an intraparenchymal and subarachnoid hemorrhage with intraventricular extension along the prior foreign body tract without evidence of hydrocephalus.

Neurosurgery recommended bedside removal of the pen due to the patient's stable GCS score and imaging showing no acute vascular process or large hematoma. Seizure prophylaxis with levetiracetam and close neurological monitoring in the ICU were maintained during removal. The procedure was performed under local anesthesia with lidocaine and procedural sedation (Versed and fentanyl). Findings afterwards included minimal bleeding with no cerebrospinal fluid (CSF) leakage. Postoperatively, the patient remained under ICU observation with hourly neurological checks and repeat imaging (Figure 2).

Following removal, the patient exhibited intermittent waxing and waning mental status and seizure-like activity, prompting an increase in levetiracetam dosage as recommended by neurology. Ophthalmology was then consulted for visual deficits in the right eye postoperatively. Examination revealed a fixed and dilated right pupil that was non-reactive to light or pain. No evidence of optic nerve swelling, and mild proptosis with minimally elevated intraocular pressure was noted (22, 21, and 23 mmHg). The findings were consistent with posterior traumatic optic neuropathy due to optic nerve involvement at the optic canal, and the overall prognosis for vision recovery was poor.

By the 11th day of hospitalization, the patient’s neurological condition had returned to his baseline status (GCS 15) with a therapeutic level of levetiracetam. Ophthalmology additionally cleared the patient due to stable visual exam findings and no concern for compartment syndrome. He remained hemodynamically stable, seizure-free, and his repeat imaging revealed no new intracranial hemorrhage and temporal resolution of prior bleed. The patient was later discharged to his facility.

## Discussion

Although a highly uncommon occurrence, penetrating orbitocranial injuries require a multidisciplinary approach. Foreign body trauma to the optic canal can result in compression or transection of the optic nerve, causing irreversible damage. Key considerations in management involving the optical canal include first-line imaging through CT scans to determine the course of the object, severity of damage, and the relationship between the foreign body and the anatomical structures it is close to [8]. In this case, utilization of the CT head helped visualize the termination of the ballpoint pen at the temporal lobe of the brain, a small subarachnoid hemorrhage along the path of penetration, and several small intracranial hematomas.

Imaging was additionally utilized to rule out potential differential diagnoses that the penetrating object may have caused. One such diagnosis that was ruled out was a potential carotid cavernous fistula by identifying if there is an enlarged superior ophthalmic vein or an enlarged cavernous sinus. This was visualized through the utilization of the CT angiography, which revealed no acute process in this case. Another consideration was for orbital apex syndrome, where an MRI was utilized to identify if there was swelling or if other cranial nerves were being affected. After identification of the severity of penetration, quick referral to surgery is important to help remove the object from the patient and prevent further worsening symptoms. Understanding of key characteristics, such as the path and termination of the penetration through imaging, was the driving force behind the decision to remove the pen at bedside. While there is a risk for fatal hemorrhage, the CT angiography being negative for an acute vascular process, the CT head showing no large hematoma or mass effect, and the patient being alert with a GCS score of 14 helped guide the decision [5,6]. While in this case the patient was neurologically alert, it is important to consider that the patient's condition could worsen immensely depending on movements and actions undertaken by both the patient and clinicians. Due to this, patients with penetrating injuries are more often taken to the operating room when considering the risks [9,10].

During the removal of a penetrating object, the utilization of anesthesia and sedation can help the patient through pain and avoid movements that add difficulty in a clean removal. In addition, the focus of seizure prophylaxis during the removal is important due to the level of intracranial hemorrhage that may occur. After removal, frequent ophthalmologic assessment of damage to the eye is imperative due to penetrations’ interactions with the optic nerve and canal and the lasting impacts left by these interactions. One such possible impact is sympathetic ophthalmia, which could lead to bilateral blindness if not correctly monitored and treated [11]. As such, the patient in this case was educated on the risk of possible occurrence and to follow up with an ophthalmologist regularly at his facility. Lastly, continuous neurological and imaging follow-up is important immediately after removal and in the long term to monitor the patient’s mental status and the impacts due to the extent of interaction of the orbital penetration with its surrounding anatomical structures, and the concern for potential worsening intracranial hemorrhage or herniation.

## Conclusions

An orbitocranial penetrating injury due to foreign objects is a rare yet severe occurrence with significant neurological and ophthalmological complications that require prompt and interdisciplinary management. This case highlights the need for early imaging evaluation to determine the course of the object and presents the rare case of bedside removal of a foreign body in a neurologically stable patient with minimal vascular injury. Although this patient had an intracranial bleed, seizures, and traumatic optic neuropathy, coordination among multiple disciplines played a key role in stabilizing the patient.
